# A Novel Fingerprint Sensing Technology Based on Electrostatic Imaging

**DOI:** 10.3390/s18093050

**Published:** 2018-09-12

**Authors:** Kai Tang, Aijia Liu, Wei Wang, Pengfei Li, Xi Chen

**Affiliations:** State Key Laboratory of Mechatronics Engineering and Control, Beijing Institute of Technology, Beijing 100081, China; tangkai01@bit.edu.cn (K.T.); 2220170090@bit.edu.cn (A.L.); wywei@bit.edu.cn (W.W.); pfli@bit.edu.cn (P.L.)

**Keywords:** electrostatic imaging, fingerprint sensing, MEMS, electrostatics field

## Abstract

In this paper, we propose a new fingerprint sensing technology based on electrostatic imaging, which can greatly improve fingerprint sensing distance. This can solve the problem of the existing capacitive fingerprint identification device being easy to damage due to limited detection distance and a protective coating that is too thin. The fingerprint recognition sensor can also be placed under a glass screen to meet the needs of the full screen design of the mobile phone. In this paper, the electric field distribution around the fingerprint is analyzed. The electrostatic imaging sensor design is carried out based on the electrostatic detection principle and MEMS (micro-electro-mechanical system) technology. The MEMS electrostatic imaging array, analog, and digital signal processing circuit structure are designed. Simulation and testing are carried out as well. According to the simulation and prototype test device test results, it is confirmed that our proposed electrostatic imaging-based fingerprint sensing technology can increase fingerprint recognition distance by 46% compared to the existing capacitive fingerprint sensing technology. A distance of more than 439 μm is reached.

## 1. Introduction

In recent years, with the continuous upgrading of smartphones and the increasing demand for online and mobile payments, biometric-based personal identification technology has the advantages of convenience and security [1,2]. Commonly used biometric methods include fingerprint recognition [3,4,5,6], iris recognition, and facial recognition [3,4]. Among them, fingerprint recognition is the most widely used biometric identification method. There are various ways to obtain fingerprints, including optical, temperature, pressure, acoustic, and capacitive methods. The optical fingerprint sensor captures a fingerprint image through the lens and can penetrate thicker glass. Synopsys’ optical fingerprint sensor can be placed at the bottom of the screen, independent of the thickness of the glass, but at a higher cost and with limited security, it can be deceived by fingerprint images [3]. A fingerprint sensor based on thermal detection uses a thermoelectric material to measure the amount of heat transferred from the sensor to the fingerprint to obtain a fingerprint image [3]. The pressure type fingerprint sensor is based on the piezoelectric effect. When the finger is placed on the top surface of the sensor, only the ridge is in contact with each sensor unit. Ultrasonic fingerprint sensors use the principle of medical ultrasound to create a visual image of the fingerprint with deep penetration [5,6]. Qualcomm’s ultrasonic fingerprint sensor can also be placed at the bottom of the screen, with a penetration capability of 700–800 μm, but at a higher cost, it is currently only available on the latest flagship phones [5]. Capacitive fingerprint sensors obtain fingerprint images by measuring the difference in capacitance between the fingerprint ridges and valleys and the sensor [7,8,9,10,11,12]. At present, the most widely used fingerprint identification tool is a capacitive sensor because it is low in cost and small in size, and it is convenient to be deployed in the system. Capacitive fingerprint sensing achieves fingerprint pattern recognition by sensing changes in capacitance caused by different distances of the ridges and valleys on the sensor surface [10,13,14,15]. Existing capacitive fingerprint sensors require high mechanical durability due to their mounting on mobile devices and their high touch operating frequency. However, due to the detection distance limitation (<250 μm) of the existing capacitive fingerprint sensor, the thickness of the protective coating of most of the current sensor-sensitive surfaces is limited to 50–100 μm [10]. Scratches and abrasion can be generated because of its thinness and insufficient coating hardness. If the fingerprint recognition distance can be effectively improved, a thicker protective coating or a certain thickness of the glass protective layer can be used to improve the life of the fingerprint sensor.

At present, the detection distance of commercial capacitive fingerprint sensors is kept at approximately 250 μm (including the sensor electrode outer layer thickness and sensor protective layer thickness or stacking relatively durable, high-quality glass on a sensor chip) [10,16,17]. In recent years, many research institutions have increased the detection distance to approximately 300 μm using circuit or structural design. An increase of the detection distance to50 μm between the pixel and the peripheral fingerprint pattern was accomplished. The edge capacitance may be larger than the capacitance formed between the pixel and the fingerprint perpendicular to the pixel, and the resolution and contrast of the fingerprint image are significantly reduced.

In order to improve the detection distance of the fingerprint sensor, we adopt a new detection principle and propose to use the fingerprint electrostatic imaging method to detect the fingerprint. At present, electrostatic detection is mainly used in the fields of electrostatic protection, lightning warnings, etc., and it is rarely used in the field of human body static detection. However, the team of the Centre for Physical Electronics and Quantum Technology (CPEQT) of Sussex University in the United Kingdom has carried out non-contact applications to the human body surface. Electrostatic imaging research [18,19,20,21] which constructed a small electrostatic imaging device for the non-contact detection of the human heart.

We used MEMS technology to design high-sensitivity electrostatic imaging arrays and analog and digital signal processing circuit structures, and we simulated and tested them. According to the simulation and prototype test device results, it is confirmed that our proposed electrostatic imaging-based fingerprint sensing technology can increase the fingerprint recognition distance by 46% compared to the existing capacitive fingerprint sensing technology. A distance of more than 439 μm is reached.

## 2. Materials and Methods

### 2.1. Electrostatic Field Distribution of Fingerprints

When the human body moves, it will be electrostatically charged for various reasons. The static charge will be concentrated in the human body’s tip. The finger is the tip of the human body. The fingerprint surface has raised ridges and depressed valleys, so a non-uniform electric field distribution is formed between the finger fingerprint and the sensor sensing surface when the finger is close to the screen or the sensor surface. Hand fingerprint image information can be obtained by detecting a non-uniform electric field distribution around the fingerprint. Table 1 shows the human body voltage values when the human body is active under different relative humidity (RH) conditions. As can be seen from Table 1, the human body voltage is basically above V [22].

It can be seen from this table that the human body always has a voltage of several hundred volts or more due to activity, and the static electricity carried by the human body can be used as a charge source for detection.

**Figure 1:**
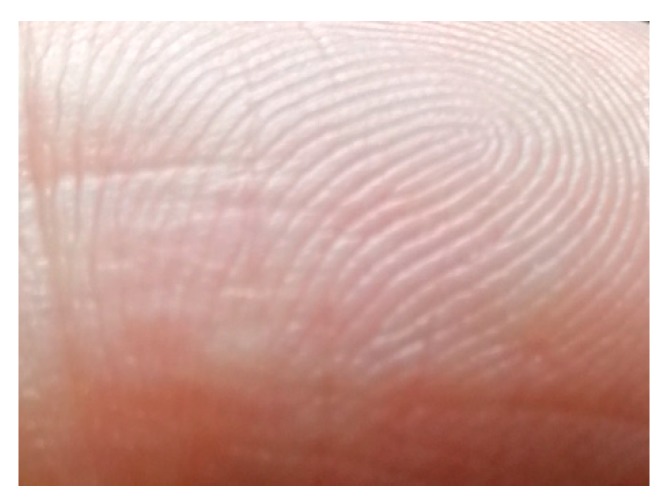
Hand fingerprint photo.

**Figure 2:**
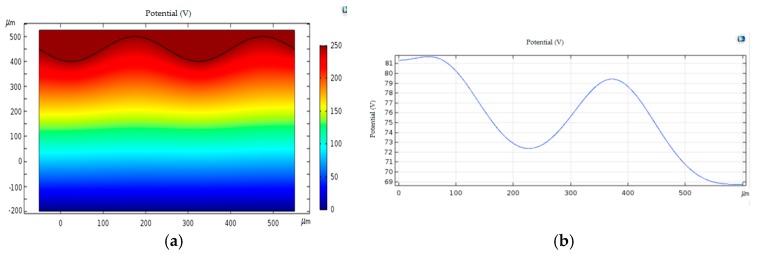
Fingerprint surface electrostatic field distribution. (**a**) the electric field distribution; (**b**) the electric field strength.

Figure 1 shows a photograph of the ridges and valleys of the hand fingerprint. It can be seen that the ridges and valleys of the fingerprint have a significant height difference. Figure 2 shows the electric field distribution of the electric field of the finger ridge and valley plane in glass. In this figure, the period of the ridge and the valley is set to 150 μm, and the depth of the valley is 50 μm. As can be seen from the Figure 2b, due to the presence of the ridges and valleys, the spatial electric field still has an uneven distribution (greater than 12 V) at a distance of 400 μm from the plane of the finger ridge. The high-sensitivity (2.3 V/m) array electric field sensor is designed to detect the spatial electric field, and a distribution image of the spatial electric field can be formed, thereby realizing the perception of the fingerprint by electrostatic imaging.

### 2.2. Principle of Fingerprint Electrostatic Imaging

**Figure 3:**
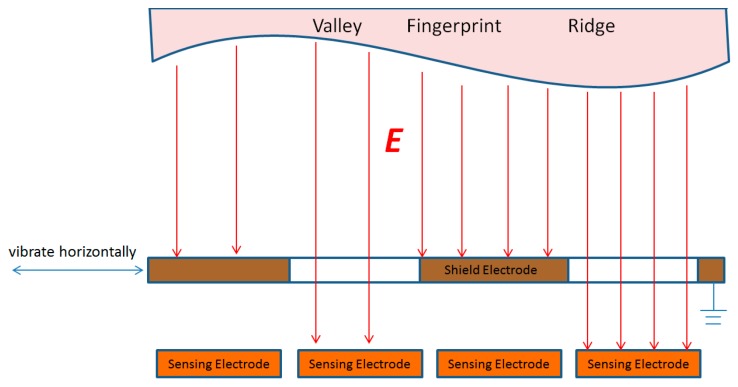
Horizontal vibration type sensing electrode array.

Electrostatic imaging detection is a method that obtains the target contour and the charged information of each part by detecting the electrostatic field in the space around the target. In order to realize the detection of the spatial electrostatic field distribution, an electrode array is needed for regional electric field induction, thereby obtaining a pixelated spatial electric field distribution. To detect the electrostatic field, it is necessary to modulate the electrostatic field to change the electric field prior, so as to form a constantly changing alternating current on the sensor electrode and realize the magnitude and direction of the electrostatic field by detecting the alternating current. This paper proposes a lateral vibration type induction electrode array to achieve electrostatic imaging. As shown in Figure 3, the electrostatic induction array is divided into two parts, the upper layer is a grounded grid-shaped shield electrode, and the lower layer is a planar detection electrode array. The grid-shaped shielding electrode can vibrate horizontally under the drive of the driving electrode. When the grid-shaped shielding electrode directly covers the sensing electrode, the shielding of the sensing electrode of the area is realized, and the detection of the sensing electrode of the adjacent area is also realized. This also led to the realization of the electrostatic field detecting modulation in the area where the electrodes are located.

The higher the frequency at which the electrostatic field is modulated, the higher the induced current generated by the electrostatic field of the same size, due to the relationship I = q/t, and the higher the sensitivity of electrostatic detection.

## 3. Sensor Design and Simulation

### 3.1. Sensor Design

The electrostatic fingerprint sensor is divided into a MEMS electrostatic sensing electrode array portion and a signal processing portion. The MEMS electrostatic sensing electrode array portion includes a protective layer, a shielding layer, and a signal sensing layer. The protective layer is made of a glass layer, and the shielding layer is made of a metal film as a shielding electrode. The sensing and signal processing layer adopt a deposited metal layer as a signal sensing electrode, and each of the two sensing electrodes constitutes a differential pair. The differential signal is amplified first-stage using a MOS circuit, and the multi-channel signal selection circuit is used for column and row signal selection. The electrostatic signal is amplified and AD converted. Finally, the digital signal is released as an output. The electrode film thickness was set to 0.2 μm.

**Figure 4:**
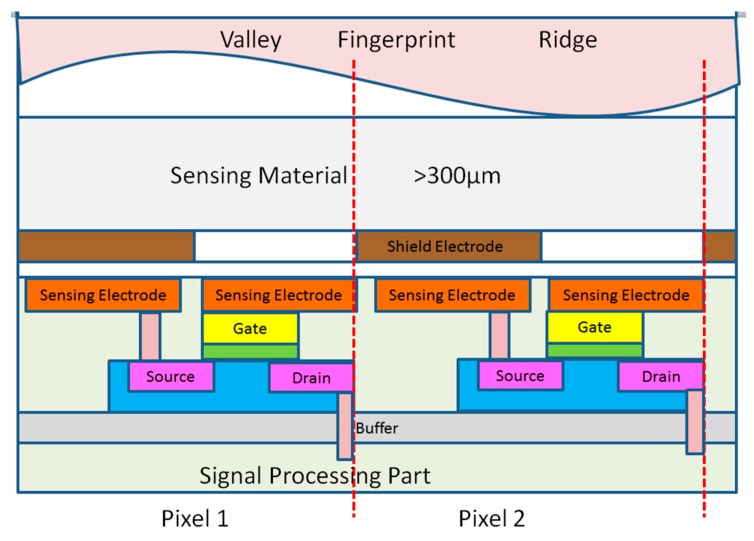
Electrostatic fingerprint sensor structure.

The electrostatic fingerprint sensor structure is designed as shown in Figure 4.

#### 3.1.1. MEMS Structure of the Electrostatic Fingerprint Sensor

**Figure 5:**
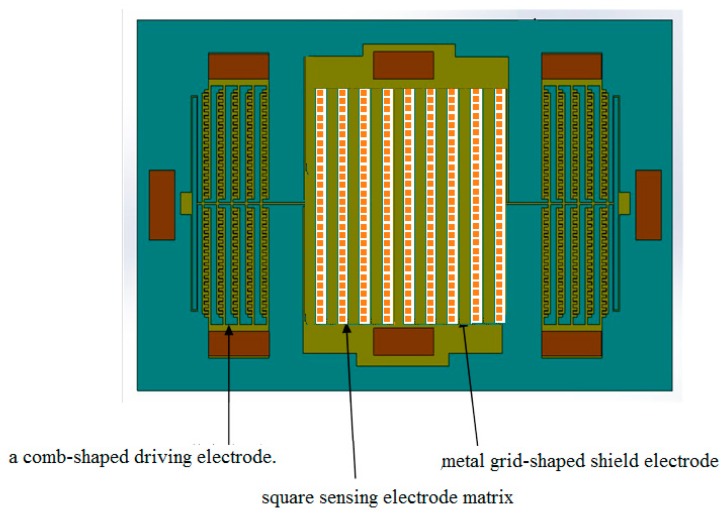
MEMS structure of the electrostatic fingerprint sensor.

The MEMS structure of the electrostatic fingerprint sensor is composed of a metal grid-shaped shield electrode, a square sensing electrode matrix, and a comb-shaped driving electrode. As shown in Figure 5.

The single crystal silicon wafer is electrically connected by electrostatic bonding and fixing to the silicon wafer. The shield electrode is a set of elongated metal grid arrays deposited on a silicon wafer. The fixed silicon comb drive electrode is in contact with the lead pads on the silicon substrate. The entire suspended silicon vibrating frame is connected to the silicon substrate through a folding beam and is connected to the ground electrode on the silicon substrate through the beam and the anchor point. Under the excitation of electrostatic force, the folded beam periodically vibrates, and the shield electrode array reciprocates with the vibrating frame. The external electric field is periodically shielded, thereby generating a periodically varying induced electric quantity on the surface of the sensing electrode on the silicon substrate. The electric field strength is obtained by measuring an induced current proportional to the electric field strength between a pair of two adjacent sensing electrodes. The dimensions of the device are as follows: the sensor unit size is 7 × 7.5 mm. The shield electrode width and spacing are 70 μm, the sensing electrode width is 50 μm, the pitch is 20 μm, and the effective overlap length of the shield is 1.5 mm. There are 80 sensing electrode pairs. The thickness of the shielding electrode and the sensing electrode are both 0.2 μm, the length of each segment of the folded beam is 900 μm, the horizontal connection between the segments is 200 μm long, the beam width is 12 μm, the distance between the shield electrode and the sensing electrode (key and suspension distance) is 3 μm, and the comb is 80 μm. The width and spacing of the tooth electrodes were both 6 μm, the number of comb teeth was 100, the thickness of the silicon diaphragm (the thickness of the comb teeth and the beam) was 50 μm, and the total mass of the vibrating portion was 0.278 mg.

The shielding electrode is driven by electrostatic excitation, and the electrostatic excitation driving method is the most commonly used driving method in the current MEMS field. The main principle is that when the capacitor is charged, the two plates have equal amounts of different charges. The different charges attract each other so that the two electrodes have a gravitational effect. When the capacitor is not energized, the gravitational force disappears. The mode of use is electrode grounding, and the other electrode is connected to the periodic square wave voltage so that the amplitude and vibration frequency of the shield electrode structure can be controlled by controlling the magnitude and frequency of the square wave voltage. According to the size, structure, and movement mode of the shield electrode, the vibration resonance point of the shield electrode is obtained at 4893 Hz. Therefore, the electrostatic drive frequency is driven by 4.9 kHz, and a higher efficiency can be obtained.

#### 3.1.2. Electrostatic Fingerprint Sensor Signal Processing Part

**Figure 6:**
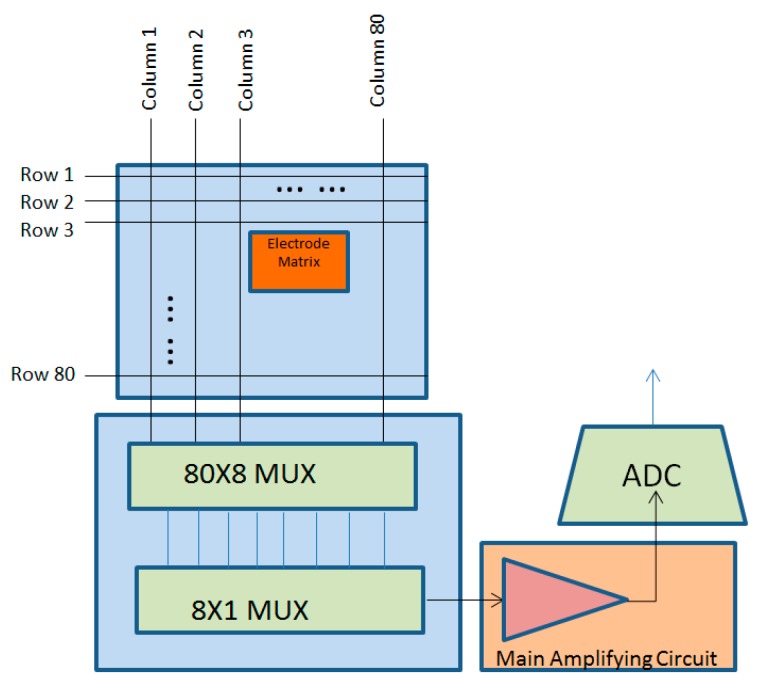
Electrostatic fingerprint sensor signal processing.

The signal processing part of the sensor is divided into four parts: IV conversion, channel selection, the main amplification circuit, and AD conversion. Each electrode signal output is performed in order, as shown in the figure. The IV conversion section uses the FET as the first stage for converting the induced current signal on the electrode into a voltage signal, and then channel selection is performed using the multi-channel data selector through row and column selection. A pair of electrode signals are selected from 80 × 80 electrodes to enter the main amplifying circuit for amplification. Then, a digital-to-analog conversion is performed by the AD circuit and, finally, the output is performed in order. The data selection section is shown in Figure 6.

### 3.2. Sensor Detection Performance Analysis and Simulation

First the sensor induced current is calculated.

When the measured electric field is perpendicular to the sensing electrode, the amount of charge change on the surface of a single sensing electrode in one cycle is Δq, the total number of sensing electrodes is *n*, the vibration frequency is *f*, and the magnitude of the induced current (*i*) of the sensor can be approximated as

$$i=\frac{dq}{dt}\approx n\cdot \Delta q\cdot f$$

The measured electric field amplitude is *E*, the spatial dielectric constant is ε, and the effective sensing area of the sensor is *A*. According to the Gauss theorem, the sensor’s induced charge is

$$q^{\prime }=\varepsilon EA$$

Therefore, the magnitude of the induced current of the sensor can be expressed as

(1) $$i=\frac{dq^{\prime }}{dt}\approx \varepsilon E\frac{dA}{dt}$$

Since the electrode area is fixed, the electrode vibration period is fixed,$\frac{dA}{dt}$ can be approximated as a constant, it can be known from Equation (1) that the magnitude of the induced current of the laterally vibrating electrostatic imaging MEMS sensor is proportional to the amplitude of the measured electric field.

When the sensor can detect that the current sensitivity is fixed, the sensitivity of the sensor to the electric field can be obtained from Equation (1)

(2) $$E\approx \frac{i}{\varepsilon \frac{dA}{dt}}$$

When we used the quartz glass with a dielectric constant ε of 3.5, an electrode size of 50 × 50 μm, a vibration frequency of 4.9 kHz, and a current detection sensitivity of 1 nA, the electric field detection sensitivity could be calculated as 0.23 V/m. Considering the noise in the circuit, we reduce the sensitivity of the electric field detection by an order of magnitude as 2.3 V/m.

**Figure 7:**
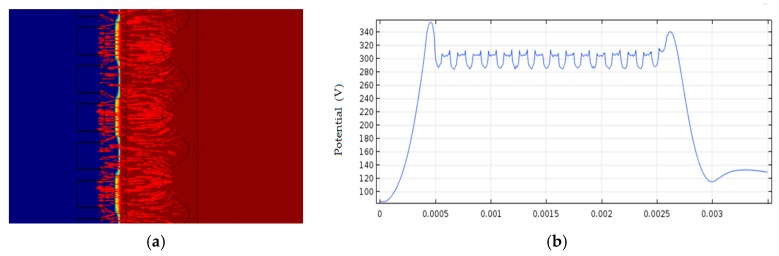
Simulation results. (**a**) electric field simulation; (**b**) the electric field strength.

The sensor structure is modeled and simulated using software, and the simulation results are shown in Figure 7. It can be seen from Figure 7 that, due to the presence of the shielding electrode, the electric field strength of the sensing electrode blocked by the shielding electrode and the electric field strength of the unobstructed sensing electrode can reach 35 V/m. The difference in electric field strength is much greater than the sensitivity of the sensor (2.3 V/m.) in the case of considering noise, so the sensor of the system has the sensitivity to meet the application requirements.

## 4. Sensor Measurement Results

**Figure 8:**
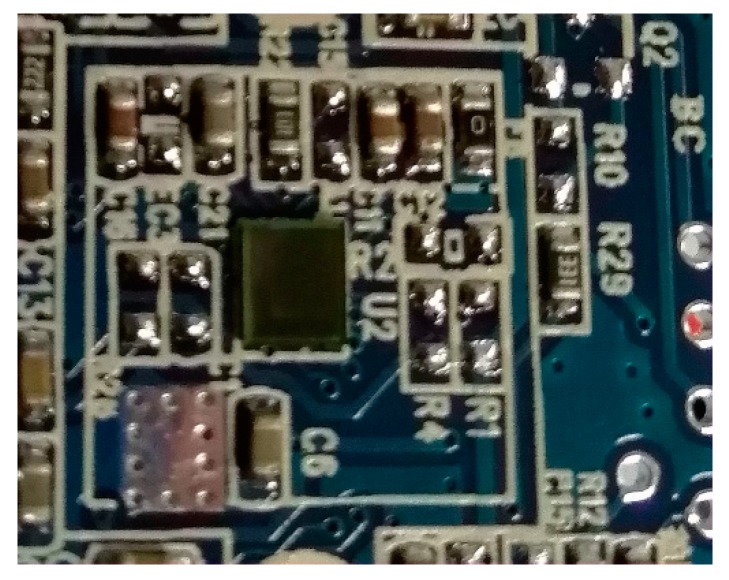
Physical prototype of a fingerprint sensor based on electrostatic imaging.

Figure 8 shows a physical prototype of a fingerprint sensor based on electrostatic imaging. The sensor is covered with a glass cover of different microscopes to realize different detection distance adjustments and so the actual fingerprint is detected.

**Figure 9:**
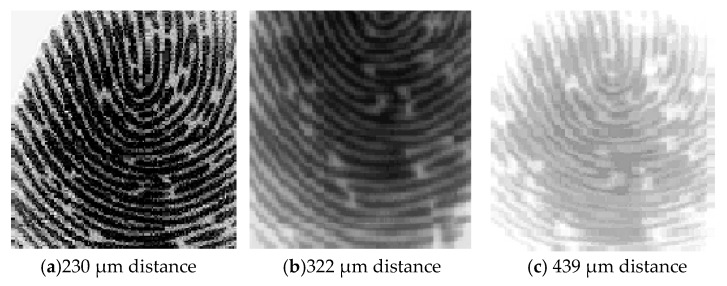
Fingerprint sensing result based on electrostatic imaging.

We used a prototype sensor to collect 50 fingerprint images at different distances for analysis. A sensing result is shown in Figure 9. Figure 9a shows the result of the sensor covered with the no. 2 coverslip. Its thickness is 213 μm. The fingerprint is 230 μm from the sensor plane and the image is very clear.

Figure 9b shows the result of the sensor covered with the no. 3 coverslip, which has a thickness of 322 μm. There is a fingerprint distance of 356 μm from the sensor plane. The image is clear.

Figure 9c shows the result of the sensor covered with the no. 4 coverslip, which has a thickness of 439 μm. There is a fingerprint distance of 456 μm from the sensor plane. The image is still recognizable.

There are some typical methods for evaluating fingerprint image quality, such as [23,24]. However, in order to compare with the performance of the new fingerprint sensors, we have used the method from [10] for evaluation, which can be compared with the sensor performance in the [10].

Performance evaluation using the method of calculating contrast in [10], the contrast calculation method uses Equation (3).

(3) $$\mathrm{Contrast}=\frac{\max\;\mathrm{of}\;\mathrm{gray}\;\mathrm{value}-\min\;\mathrm{of}\;\mathrm{gray}\;\mathrm{value}\;}{\max\;\mathrm{of}\;\mathrm{gray}\;\mathrm{value}+\min\;\mathrm{of}\;\mathrm{gray}\;\mathrm{value}}$$

**Figure 10:**
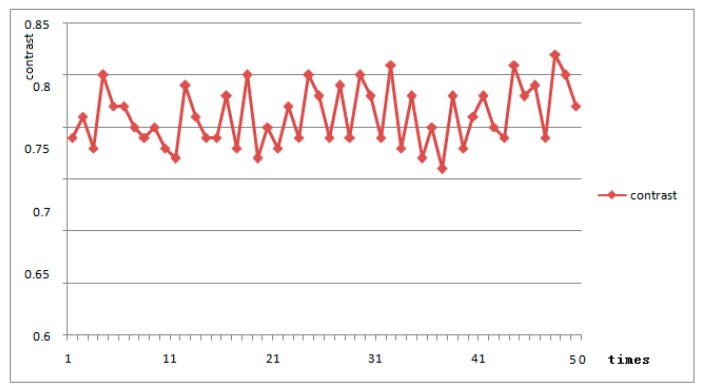
Fingerprint imaging contrast.

Contrast analysis of 50 sets of 439 μm distance images, the results shown in the Figure 10, the average contrast is 0.81. This far exceeds the traditional capacitive fingerprint identification method. With a contrast ratio of 0.59 at 300 μm, the detection distance is increased by 46% compared to the conventional capacitive fingerprint recognition method, with better contrast.

At a distance of 322 μm, the contrast of the system imaging is 0.99. Compared to the [10] method, the detection distance is increased by 7% with the same contrast.

Therefore, the fingerprint sensing technology based on electrostatic imaging can have better performance at a distance of 439 μm than the advanced capacitive fingerprint recognition mentioned in [10] at a distance of 356 μm.

## 5. Conclusions

In this article, we propose a new electrostatic imaging-based fingerprint sensing technology based on the principle of electric field detection, which has a farther detection distance than the existing capacitive fingerprint sensing technology. Based on the analysis of fingerprint electrostatic distribution and electrostatic imaging technology, we designed the MEMS sensor structure, an electrode size of 50 × 50 μm, work on vibration frequency of 4.9 kHz, and forming an 80 × 80 detection array. The corresponding signal processing circuit was designed. The detection signal amplification, AD sampling and data output are realized by means. Simulation analysis and experimental verification were carried out by the multiplexed circuit. The results show that the prototype test device has a detection sensitivity of 2.3 V/m. Fifty fingerprint imaging experiments covering different thicknesses of coverslips were carried out. The experimental results show that the detection distance of our sensor is 46% higher than the distance of traditional capacitive fingerprint recognition with better imaging quality, and it is increased by 7% compared with the detection distance of advanced capacitive fingerprint identification technology proposed by [10]. We believe that the new fingerprint sensing technology based on electrostatic imaging can improve the detection distance of existing capacitive fingerprint sensors, increase the thickness of the protective layer, and improve the life of the sensor. In the case of implementing a transparent detecting electrode, it is used as an in-display fingerprint sensor, and has the advantages of low cost and high safety.

## Figures and Tables

**Table 1:** Human body activity voltage (V) [22].

Human Activity Mode	RH: 10–20%	RH: 65–90%
Walking on a synthetic carpet	35,000	1500
Walking on a plastic carpet	12,000	1250–1750
Working on the workbench	6000	1000
