# Correction: Tang, K., et al., A Novel Fingerprint Sensing Technology Based on Electrostatic Imaging. *Sensors* 2018, *18*, 3050

**DOI:** 10.3390/s19071725

**Published:** 2019-04-10

**Authors:** Kai Tang, Aijia Liu, Wei Wang, Pengfei Li, Xi Chen

**Affiliations:** State Key Laboratory of Mechatronics Engineering and Control, Beijing Institute of Technology, Beijing 100081, China; tangkai01@bit.edu.cn (K.T.); 2220170090@bit.edu.cn (A.L.); wywei@bit.edu.cn (W.W.); pfli@bit.edu.cn (P.L.)

The authors wish to make the following corrections to this paper [1]:

We have found one inadvertent error in our paper published in this journal [1]: 

At the beginning of Section 4, “Sensor Measurement Results”, we missed a paragraph as follows:

The prototype of the Micro-electromechanical Systems (MEMS) and Integrated Circuit (IC) composite structure requires a long lifecycle and a high cost. The Charge Coupled Device (CCD) chip has a charge coupling and amplification function, so the electrostatic imaging principle was verified by using the modified CCD chip. By removing the filter on the CCD surface, a prototype of a fingerprint sensor based on electrostatic imaging was built. In a dull environment, the modified CCD chip generates an induced charge on the electrostatic field through the surface pixel and uses the charge amplifying circuit to perform induced charge amplification to realize the electrostatic imaging function. Compared with the sensor designed in the third section of the thesis, the modified CCD chip has fewer shield electrodes and can only achieve single-shot electrostatic imaging; however, it can verify the electrostatic imaging principle and design scheme proposed in the paper.

This change has no material impact on the conclusions of our paper. We apologize to our readers.
